# Mini Review: linkages between essential tremor and Parkinson’s disease?

**DOI:** 10.3389/fncel.2013.00118

**Published:** 2013-07-31

**Authors:** Yiwen Wu, Jianqing Ding, Yuan Gao, Shangdi Chen, Li Li, Rena Li

**Affiliations:** ^1^ Department of Neurology and Institute of Neurology, Ruijin Hospital, Shanghai Jiao Tong University School of MedicineShanghai, China; ^2^ Department of Health and Kinesiology, Georgia Southern University, StatesboroGA, USA; ^3^ Center for Hormone Advanced Science and Education (CHASE), International Biomedical Research Training Program (IBRTP), Roskamp InstituteSarasota, FL, USA

**Keywords:** essential tremor, Parkinson disease, brain imaging, genetic markers, tremor frequency, intensity

## Abstract

Essential tremor (ET) and Parkinson’s disease (PD) are two of the most common movement disorders. Tremors are the primary symptoms of ET and of some PD patients, the two are often mistaken for each other. Especially since there are no available differentiate tests for the tremor of ET or PD, the early diagnoses mainly based on clinical assessments of medical symptoms, family and medication history, and examination by physicians. There is increasing evidence suggesting an association between ET and PD, such as a similar tremor frequency, overlapping resting tremors (a typical PD tremor), postural tremors (mainly in ET patients) in both ET and PD patients, and many ET patients develop PD later in life. Although it is difficult to make a differential diagnosis of ET and tremor-dominant PD based on clinical assessment, recent developments of objective measurements, such as brain imaging, neuropathology, and genetic analysis, has opened a helpful window for distinguishing ET from PD. In this mini review, we included literatures of ET and PD studies and discussed various advanced methods for differential diagnosis between ET and PD such as neuroimaging, genetic markers, tremor intensity and frequency, and drug-responses.

## Introduction

Tremor is an involuntary trembling of the body or limbs. In general, tremor can be classified as “physiological tremor” and “pathological tremor”. The physiological tremor is a rapid transient tremor of extremely low amplitude found in the limbs and sometimes the neck or face of healthy individuals. The physiological tremor usually is not noticeable and can only be subtly detected by an electromyogram (EMG) with a frequency range of 8–10 Hz in most of cases (Freund and Dietz, 1978; Henneman, 1979). While the cause of physiological tremors is unknown, noticeable physiological tremors are often increased with ageing and enhanced by anxiety, fatigue, low blood sugar, use of caffeine, alcohol, and several medications (Louis et al., 2000). Pathological tremors usually have the frequency of the contractions in the range of 4–8 Hz. The most common types of pathological tremors are observed in people with essential tremor (ET), Parkinson’s disease (PD), cerebellar tremor, and dystonic tremor (Elble and Randall, 1976; Deuschl et al., 1998). Pathological tremors, such as ET and PD, are also commonly observed in aged populations, without significant gender difference (Benito-León et al., 2003; Barbosa et al., 2013). The differential diagnosis of these tremors is important since the treatment will depend on the specific etiology of each tremor type.

## Phenotypes of ET and PD

There are several types of tremors that are commonly classified by clinical features, such as resting, action, and posture tremors (Jankovic and Fahn, 1980). Some of the tremors are known to be associated with specific movement disorders. For example, patients with ET mostly suffer from a disabling postural and action tremors. A typical example of an action tremor is a tremor that gets most prominent whenever an ET patient tries to use their hands to do something and ceases typically at rest. The most recognized and characterized tremors of ET is mainly involve the hands and forearms while some ET patients also suffer from head and chin tremors (Louis, 2005; Alonso-Navarro et al., 2008). The neuropathology of ET is uncertain, although studies found that the cerebellum, brainstem or thalamus is probably involved (Deuschl et al., 2000; Kronenbuerger et al., 2007; Passamonte et al., 2012; Pedrosa et al., 2012). In PD patients, there is a predominance of resting tremors that occurs in a body part that is not voluntarily activated and is completely supported against gravity. The resting tremor usually vanishes with the initiation of a movement. However, the tremor disappears when the limbs are in extreme rest, such as when the patient is sleeping. Based on tremor type and frequency, the PD tremor can also be classified into three phenotypes, such as Type I characterized by same tremor frequency in resting and action tremors, type II is different tremor frequency in resting and action tremors, and type III is purely action tremors including postural tremor (Deuschl et al., 1998). Because of overlap of resting and action tremors in PD patients, it is easy to misdiagnose between ET and PD (type II and III).

## Association between ET and PD

Although ET and PD appear to be two distinct diseases, there is increasing evidence suggesting an association between ET and PD. The association between the two movement disorders includes a similar tremor frequency range of 4–8 Hz in both ET and PD (although 7–15 Hz tremor frequency is also reported in ET), overlapping resting tremors (a typical PD tremor) and postural tremors (mainly in ET patients) in some of ET and PD patients, and many ET patients develop PD later in life (Simões et al., 2012). Recent case–control and genetic epidemiological studies indicate that ET is associated with an increased risk of PD. For example, 5.9 to 7.1% of PD patients had previously been diagnosed with ET at least 3–5 years before the onset of PD (Louis and Frucht, 2007; Tan et al., 2008). The individuals with family history of postural tremors or ET also showed higher risk of PD (Lang et al., 1986; Zorzon et al., 2002; Spanaki and Plaitakis, 2009). In addition, functional neuroimaging studies also demonstrated association between ET and PD. Studies of dopamine transporter ligands using ^125^I-beta CIT and ^123^I-ioflupane have shown dopaminergic deficiency in the striatum of ET patients compared to controls (Isaias et al., 2008, 2010). A reduction of dopamine uptake was also reported in the putman of ET patients (Brooks et al., 1997). Moreover, studies also found PD neuropathological marker, Lewy bodies, in the locus coeruleus of some ET patients (Louis et al., 2007). However, the specific pathophysiology of the tremors in both diseases requires further investigation.

## Differential diagnosis of ET and tremor dominant PD patients

There is no biomarker for to differentiate the tremors of ET and PD except by clinical assessment. The difficulty with diagnosing early PD has been highlighted in several recent clinical trials, especially with relatively high clinical diagnostic error rates for PD and ET. As reported from multiple clinical studies, about 30–50% of ET patients are misdiagnosed as “false” ET, PD or no ET (Schrag et al., 2000; Jain et al., 2006; Quinn et al., 2011). While there is no concrete evidence on why there is such poor accuracy in diagnosing ET, it is possible that a certain subtype of ET may be involved in clinical investigations. For example, a subset of ET patients that developed PD (ET-PD) over time may express different clinical characteristics and neuropathological features compare to stable ET patients who do not develop PD. A recent study showed that ET-PD patients had fewer widespread postural and/or action tremors or cerebellar signs and required fewer ET medications than stable ET patients (Simões et al., 2012). In addition, it is pretty difficult to diagnosis ET when the patients have overlapping tremors with PD, such as resting tremors. Studies showed 19% of ET patients have rest tremors and rigidity while sustained posture tremors are also found in some PD patients (Louis et al., 2001; Cohen et al., 2003; Dotchin and Walker, 2008). Since ET is reported as a risk of PD and often occurs several years prior to the onset of PD, increasing interest in developing objective measurements, such as brain image and genetic analysis, is critical for early diagnosis of ET and the prevention of PD. 

## Current approaches for diagnosis of ET and PD:

There has been considerable interest in neuroimaging, genetic markers, tremor intensity and frequency and drug-response for diagnosis of ET and PD. Although there is no sufficient evidence for the causes of ET and tremors in PD, we have listed some commonly used methods for the diagnosis of ET and PD.

(1) Neuroimaging is currently used to provide objective measures of dysfunction in the brain in ET and PD, such as positron emission computed tomography (PET), and magnetic resonance imaging (MRI). ***[^18^F] fluorodeoxyglucose (FDG)-PET*** imaging, which assesses brain metabolism and synaptic activity, has demonstrated increased pallidothalamic and pontine activity associated with a relative reduction in cortical motor regions in PD (Eidelberg et al., 1997; Wang et al., 2013). A number of imaging studies indicated clear differences between ET and PD in terms of basal ganglia involvement, such as the striatal ***[^123^I]beta-CIT binding*** ratios in ET were within normal ranges while reduced specific binding was evident in the putamen and caudate nucleusin PD (Asenbaum et al., 1998). Using ***electrode implantation*** by stereotactic cranial computed tomography (CT) and stereotactic high resolution MRI as reference image, studies measured intraoperatively thalamic local field potentials simultaneously with EMG in ET and PD patients and found that within the ventral lateral posterior nucleus of the thalamus (VLp) individual tremor-related electrophysiological signatures exist in ET and PD tremors (Pedrosa et al., 2012). However, the analyzed tremor-related local field potentials activity in the VLp is a surgical method, which limits its application in many clinical practices, except when performing deep brain stimulation (DBS), an effective treatment for ET and PD (Koller et al., 2000; Lyons et al., 2001; Rehncrona et al., 2003; Blomstedt et al., 2007). The ^123^I-ioflupane ligand with single-photon emission CT was recently approved in the United States to aid in PD diagnosis. While neuroimaging biomarkers have been extensively utilized in PD (Wang et al., 2013), appropriate use of ***^123^I-ioflupane scan*** in cases of possible ET with marked asymmetry or other Parkinsonian features is recommended and would help in detection of tremulous PD cases or cases of PD with additional ET (Bajaj et al., 2013).

(2) Genetic approaches are the next steps for diagnosis of ET and PD. Although the gene accounting for the majority of ET patients has not been identified, twin studies suggested a large role of genes over environment in ET by doubling concordance in monozygotic than that in dizygotic twins (Tanner et al., 2001; Lorenz et al., 2004) and the difference of concordance rates between monozygotic and dizygotic twins were increased in aged population (Deuschl et al., 1998). Three distinct loci were identified with linkage to ET as ETM1, ETM2 and ETM3 located at chromosome 3q13, 2p24 and 6p23 regions, respectively (Gulcher et al., 1997; Illarioshkin et al., 2002; Shatunov et al., 2006). However, most of the ET loci vary with regions, such as ETM2 was highly linked to ET in American, Spanish and Korean cohorts, but negatively linked in Czech, Italian, Latvian, Singaporean, German, Danish and French cohorts (see review by Testa, 2013). PD is the result of a loss of dopamine in the substantia nigra. Several studies demonstrated genetic markers for PD, such as a-synuclein (SNCA), MAPT, leucine-rich repeat kinase 2 gene (LRRK2), DJ-1, PARK16–18, (Mata et al., 2011). Recent studies also showed that genetic variants of leucine-rich repeat and Ig domain containing 1 (LINGO-1) gene could be risk factor for ET (Zhou et al., 2012) and polymorphism of leucine-rich repeat and Ig domain containing 2 (LINGO-2) is associated with risk of PD (Su et al., 2012). However, there are no genetic studies on ET-PD patients which may be important in identifying any genetic features of the subset ET-PD patients from stable ET patients and provide some insight on understanding the genetic association between ET and PD.

(3) Drug response is a clinical method to measure the patient’s response to a specific drug treatment to confirm the proposed diagnosis. For example, if the cause of a tremor is PD, it may respond well to medication such as levodopa or other dopaminergic medications. The limitation of using patient drug response as a confirmation of clinical diagnosis is also obvious. Studies showed that patients at early stage of PD with prominent tremors may not response to L-dopa or other dopaminergic medicine well; the medication response may not confirm the clinical diagnosis of early PD (Marjama-Lyons and Koller, 2000). Furthermore, about 30% of ET patients do not respond to the medication propranolol, the most widely accepted treatment for ET, while improvement was reported in PD tremors (Koller and Herbster, 1987; Koller and Vetere-Overfield, 1989). Alone, drug-response might not be very beneficial for differential diagnosis of ET and PD.

(4) Tremor intensity and frequency analysis is another interesting tool for differential diagnosis of ET and PD. Studies examined asymmetry of tremors on the hands of PT and ET patients, and demonstrated that the intensity of tremors was significantly asymmetric in both diseases while the frequency (number of repetitions per second) were more symmetric in ET and asymmetric in PD (Farkas et al., 2006). The different type of tremors between ET and PD were also reported by independent investigations. In general, ET starts with low amplitude tremors and gradually develops to disabling tremors. The tremor frequency may decrease while tremor amplitude (magnitude/strength) may increase as ET progresses (Mostile et al., 2012). Studies found that intensity of tremors were markedly attenuated during walking relative to resting ET patients and the intensity and frequency of Parkinsonian tremors were increased while walking than while resting (Uchida et al., 2011). However, ET resting tremors can’t be differentiated from PD resting tremors both clinically or by detecting the intensity and frequency of tremors. The relationship between tremor intensity or frequency in ET and PD is controversial. An inverse correlation between tremor intensity and centre frequency was found in ET patients while patients with PD showed only significant correlation between centre frequency and the hand with more severe tremor, not in the other hand (Machowska-Majchrzak et al., 2012). Other studies reported a significant inverse correlation between tremor intensity and centre frequency in both hands in patients with PD (Farkas et al., 2006). Further investigation on developing differential diagnosis on tremor intensity and frequency between ET and PD is needed.

## Conclusion

Although ET and PD differ in etiology and are characterized with distinct symptoms, misdiagnosis between the two is still common due to lacking of objective differential measurement. Identifying different pathological tremors in ET and PD would not only help differential diagnosis of ET and early stage of PD, but plays critical role in developing specific therapeutic strategy and treatment for the two major movement disorders. Especially, as ET has been reported as a risk of PD and often occurs several years prior to onset of PD, developing noninvasive and sensitive objective measurements is critical for early diagnosis of ET and prevention of PD. 

## Conflict of interest statement

The authors declare that the research was conducted in the absence of any commercial or financial relationships that could be construed as a potential conflict of interest.
